# Supplementary figures and images for: Comparative Analysis of the Chloroplast Genomes of Eight Species of the Genus Lirianthe Spach with Its Generic Delimitation Implications
Source: Int J Mol Sci. 2024 Mar 20;25(6):3506. doi: 10.3390/ijms25063506 (PMC10970879; doi:10.3390/ijms25063506)

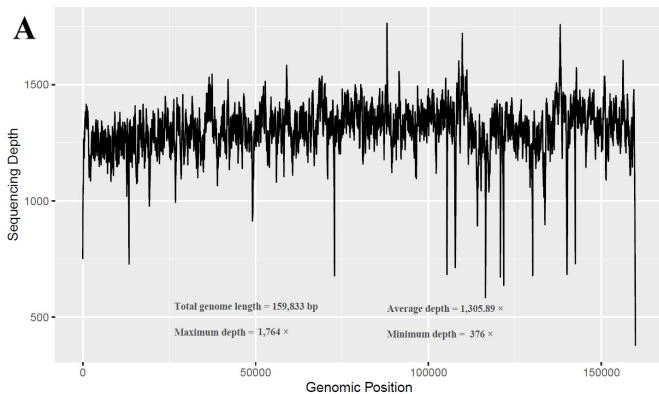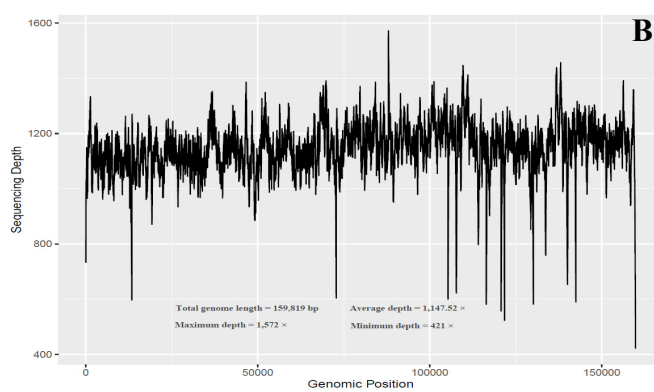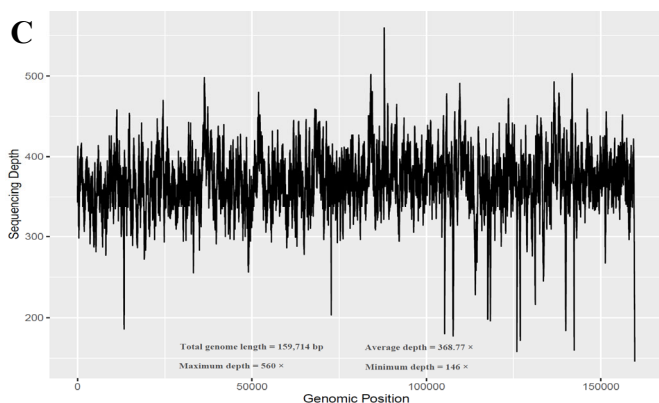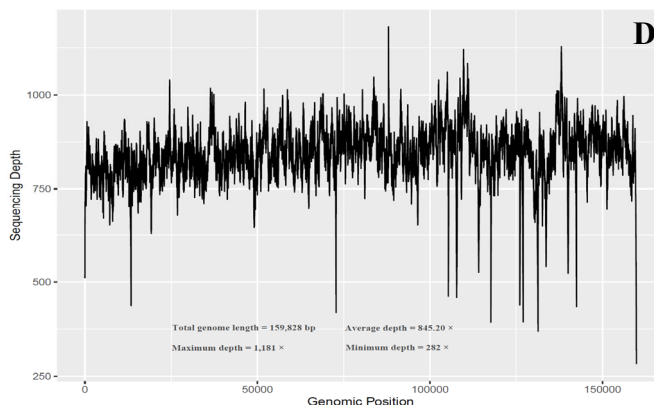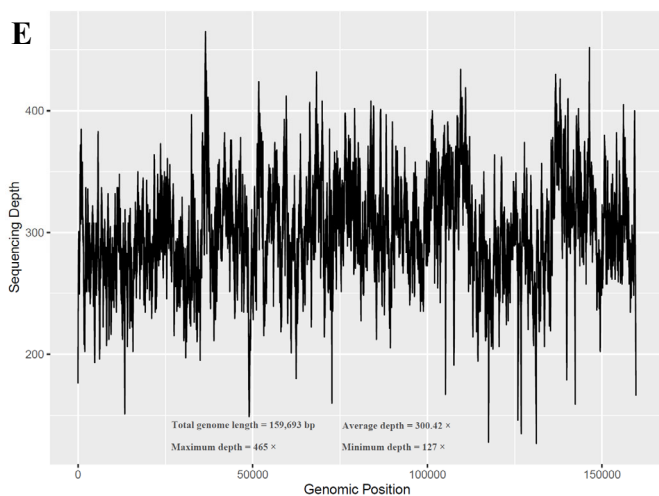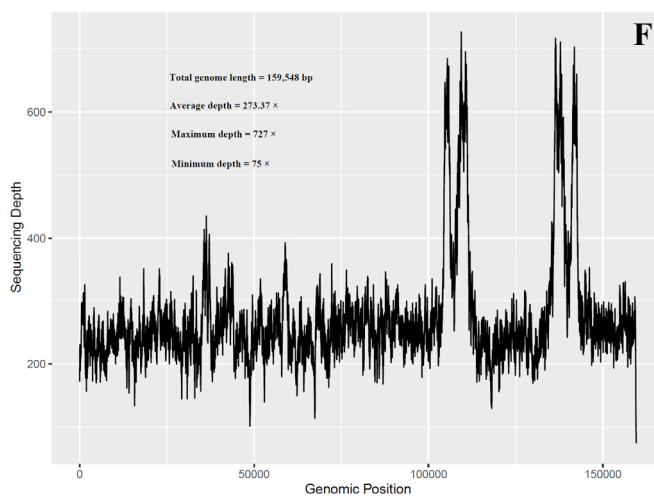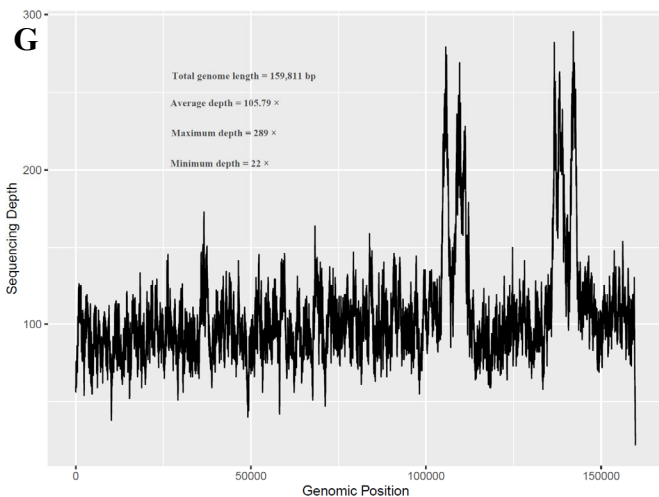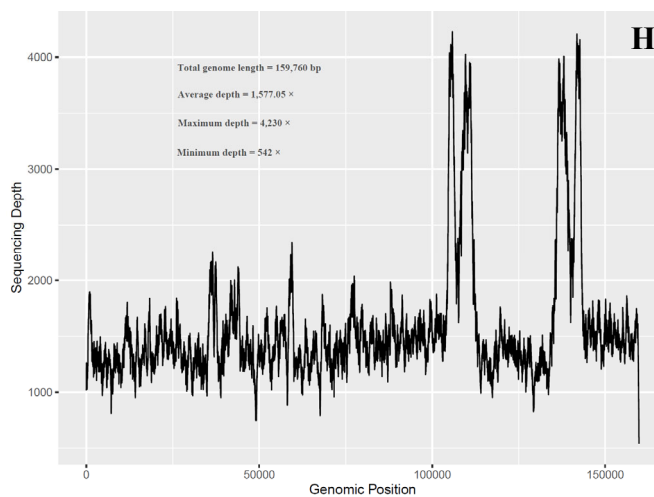

Supplement: Supplementary file 1 [file ijms-25-03506-s001.zip › Figure S1. The sequencing depth and coverage map calculated using minimap2+samtools in eight Lirianthe species.pdf]

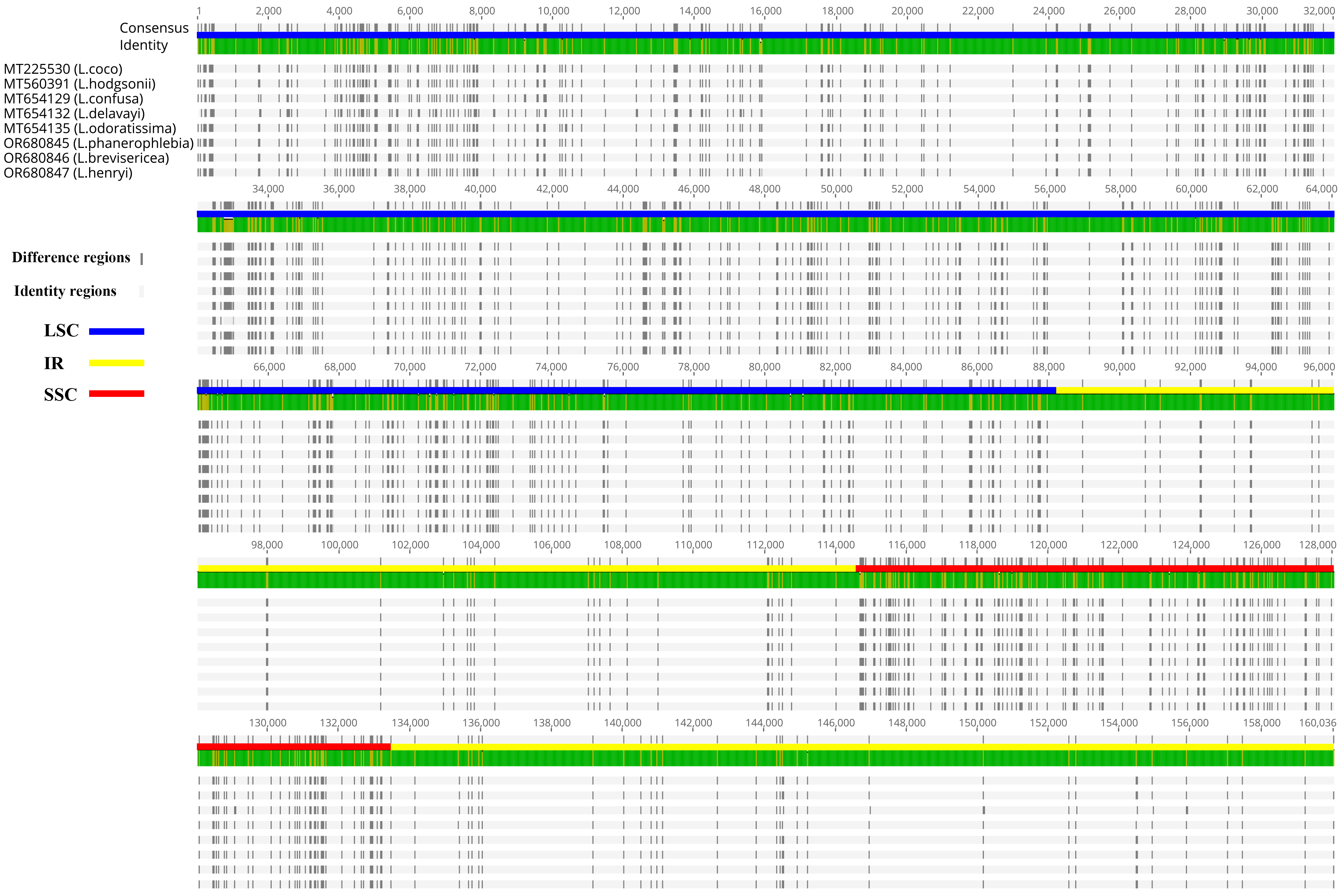

Supplement: Supplementary file 1 [file ijms-25-03506-s001.zip › Figure S2. The visualization of sequence alignment results in eight Lirianthe species.jpg]

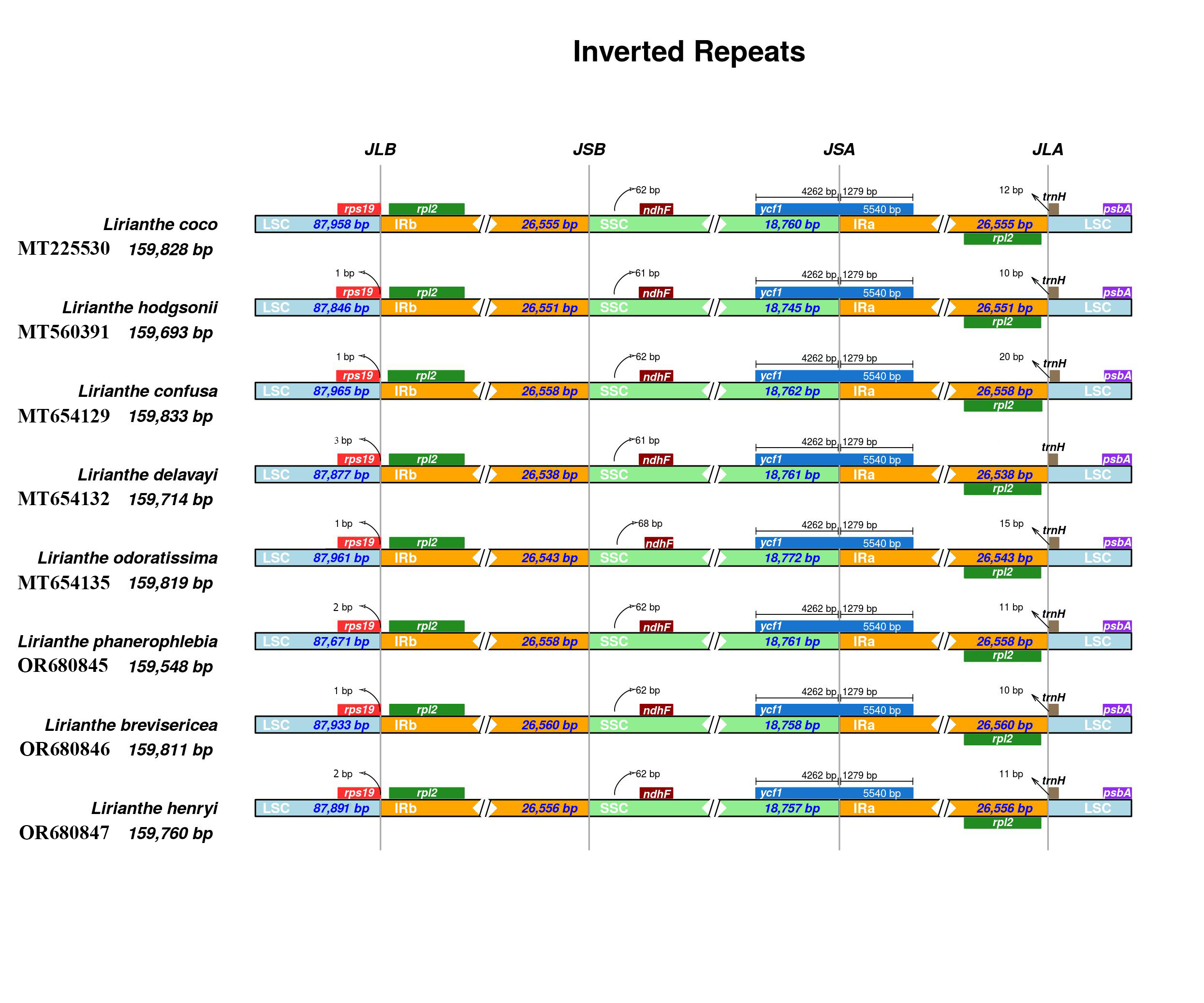

Supplement: Supplementary file 1 [file ijms-25-03506-s001.zip › Figure S3. Comparison of IR boundary in eight Lirianthe CPGs. The arrow indicated the number of base pairs that represented genes moving away from a particular region of the chloroplast genomes.jpg]
